# Exploring the impact of ineligibility on individuals expressing interest in a trial aimed at improving daily functioning regarding perceptions of self, research and likelihood of future participation

**DOI:** 10.1186/s12874-021-01464-x

**Published:** 2021-11-27

**Authors:** Christopher P. Dwyer, Helen McAneney, Fionnuala M. Rogers, Robert Joyce, Sinéad M. Hynes

**Affiliations:** 1 Applied Psychology, School of Social Sciences, Technological University of the Shannon, Athlone, Ireland; 2 School of Nursing, Midwifery and Health Systems, University College Dublin, Dublin, Ireland; 3 School of Health Sciences, National University of Ireland, Galway, Ireland

## Abstract

**Background:**

Eligibility guidelines in research trials are necessary to minimise confounds and reduce bias in the interpretation of potential treatment effects. There is limited extant research investigating how being deemed ineligible for such trials might impact patients’ perceptions of themselves and of research. Better understanding of the impact of patient ineligibility could enhance design and implementation of future research studies.

**Methods:**

Eight semi-structured telephone interviews were conducted to explore the impact of ineligibility on self-perceptions; perceptions regarding the nature of research; and the likelihood of expressing interest in future research. Data were collected and analysed thematically through inductive, interpretive phenomenological analysis (IPA).

**Results:**

Five themes emerged regarding the experience of being deemed ineligible: (1) Being deemed ineligible is emotion and reaction evoking; (2) ‘Doing your bit’: Helping others and increasing the value of research; (3) Communication of ineligibility; (4) Appreciation for those who express interest; and (5) Subsequent perceptions and attitudes towards research.

**Conclusions:**

The results suggest that being deemed ineligible can elicit negative emotional outcomes but is not likely to change perceptions of or attitudes towards research, possibly due to a desire to help similar others. Ineligibility can impact future participation in some cases, thus reducing the recruitment pool for subsequent research studies. Recommendations are provided to help minimise this risk. Advising of ineligibility in a personal way is recommended: with enhanced clarity regarding the reasoning behind the decision; providing opportunities to ask questions; and ensuring that appreciation for the patient’s time and interest are communicated.

## Background

For the purpose of scientific rigour, eligibility guidelines are necessary for research trials, as they diminish the likelihood of confounds that may impact the ability to confidently observe potential treatment effects. Consistent with this rationale, it is often the case that once a participant is deemed ineligible, they are no longer observed in the trial. Though research has been conducted on ineligibility with respect to rates [1, 2] reasons for ineligibility [3, 4], impact on achieving adequate sample sizes [5], processes for dealing with ineligible participants who have been inadvertently randomised [6, 7], and the relationship between cohort representativeness and the acquired sample [3], various literature searches yielded no research that has examined the impact of being *deemed* ineligible.

While most clinical studies exclude individuals based on pre-set criteria, there is a lack of clarity regarding how this protects the patients; and though such criteria are based on clinical reasoning, it can be argued that they may be in place more so to protect the treatment than the patient [8]. Of course, the fidelity of research trials is vital to ensuring the development, assessment and subsequent large-scale provision of potentially beneficial treatments; however, better understanding of individuals ineligible for such trials may enhance the design and implementation of future research [1].

Specifically, in the context of individuals with a chronic illness (e.g. multiple sclerosis [MS]), who expressed interest and/or consented to take part in a trial aimed at treating their illness, little is known about the effects of being deemed ineligible, with respect to their perceptions regarding themselves and the nature of research more generally. Being deemed ineligible may have negative impacts on how patients perceive: themselves, for example, ‘without use’ or being ‘denied’ potentially beneficial treatment (based on information collected from them, the severity of their condition or any other eligibility criterion); as well as the nature of research, with respect to, for example, its usefulness in real-world settings or even the likelihood of such individuals participating in other research studies.

Given the lack of extant research in this area, the aims of this study-within-a-trial^1^* (SWAT; see Footnote 1) were to explore: the impact of ineligibility on patient’s perceptions of themselves, the nature of research and the likelihood of their expressing of interest in future research studies; methods for enhancing communication with patients about their ineligibility; and strategies for enhancing recruitment in research trials, more broadly. Notably, the aims of this SWAT align with guidelines proposed by Treweek et al. [9, 10] for identifying the necessity of a SWAT. This SWAT further aimed to inform the currently ‘weak’ evidence base available for informing routine research trial decisions [10], such as, how best to recruit participants and utilise eligibility criteria.

## Methodology

### Study design & analysis

Qualitative interview data were collected and analysed thematically through an inductive, interpretive phenomenological analysis (IPA) methodology [11, 12], given its consistency with the SWAT’s focus on a homogenous group of individuals (i.e. people with MS) who share experience of a common life phenomenon [13] – being deemed ineligible for the host trial. Specifically, IPA entails a detailed account of individual lived experience, providing an idiographic framework consistent with building an understanding of experiences of the shared phenomenon among a small sample, while elucidating analyst interpretation [13, 14]. IPA involved two phases, consistent with Smith et al.’s [ 14] analysis framework: 1) each individual’s experiences and accounts were detailed, alongside the researcher’s meaning-making from an ‘insider’ perspective; followed by (2) an interpretative analysis from an ‘outsider’ perspective [15], through an iterative, recursive process, characterised by initial transcript notation, continual re-reading of the data, transcript notation, data coding and thematic identification (e.g. development of categories/themes and hierarchical ordering). Notably, reflexivity was engaged with respect to attending to such ‘reflexive echoes’ [16] following initial theme identification, across further consideration of relationships among these, further theme development and subsequent hierarchical ordering. Reflexivity engagement was conducted in order to account for the researcher’s dual role as voice for the participant and data analyst who, only naturally, engages their own experience and previous knowledge during the process [17]. In order to minimise the potential impact of related bias, member-checking was engaged (see Procedure below) and the researcher approached both interviews and analyses with an open-mind, allowing the data to ‘tell their own story’. In a practical sense, this was further facilitated by the lack of extant research in this area, as well as both the exploratory nature and inductive approach to the SWAT.

### Participants

Sixty-two individuals were deemed ineligible for the host trial. Among the most common reasons for ineligibility were: scoring below the threshold for cognitive difficulty on the Multiple Sclerosis Neuropsychological Screening Questionnaire (MSNQ; 72% of cases of ineligibility), having a co-morbid neurological condition (8.1%) and having a mental health condition (4.8%). Additional frequencies are presented in Table 1, as are all the eligibility criteria for the host trial and the rationale for each of their inclusions. Purposeful sampling was used to select participants for this SWAT. Specifically, eight categories of ineligibility (i.e. in light of criteria presented in Table 1) were identified from the ineligible cohort, including (1) experiencing an actively relapse, (2) having neurologic history other than MS, (3) having a mental disorder, (4) currently participating in another cognitive rehabilitation program, (5) institutionalisation, (6) not being a resident of Ireland, (7) not having cognitive difficulties (i.e. not severe enough for inclusion – scoring less than 22 on the MSNSQ) and (8) being deemed ineligible for more than one distinct reason. The sample size of eight was chosen in light of this, consistent with guidelines provided by Smith [12]. One individual from each category was randomly selected to be invited to interview. Though ineligible for differing reasons, the cohort was homogenous, given that all members were people living with MS in Ireland, who expressed interest in participating in the host trial, who were otherwise eligible to participate apart from one criterion (or two – as in one case), but nevertheless, deemed ineligible. Consistent with the aforementioned impact of being deemed ineligible, it was reasonably speculated that interviewing individuals who were ineligible for differing reasons would better facilitate a more informative and richer account of interviewees’ perceptions of themselves and the nature of research – which would likewise, provide the researcher conducting the analysis a similarly rich perspective. In the event that an individual was unavailable or declined to participate, another individual was randomly selected from the same category and so forth, until representation from that category ceased. Thus, a maximum of eight individuals would participate in the telephone interviews, which is within an acceptable range for best IPA practice guidelines [12].

**Table 1:** Host trial eligibility criteria

Criterion	%	Rationale
Aged 18 years or over;	0%	The content is not suitable to people under 18; for example, given the program’s focus on daily functioning associated with work, university, parenting, etc. Coupled with consideration of MS demographics, inclusion of persons 17 or under is not warranted.
Fluent in written and spoken English;	1.6%	Given the program’s focus on group interaction, fluency in the majority language (i.e. English) is vital for being able to fully participate and engage in the program, as well as avoiding confound.
Have a diagnosis of multiple sclerosis;	0%	COB-MS is a Cognitive Occupation-Based program for people with MS and cognitive difficulties; thus, a diagnosis of MS is fundamental.
Have cognitive difficulties;	72%	COB-MS is a Cognitive Occupation-Based program for people with MS and cognitive difficulties; thus, having cognitive difficulties is fundamental.
No neurologic history other than MS (e.g. dementia);	8.1%	Presence of another neurologic condition could potentially confound the research.
No history of major depressive disorder, schizophrenia, or bipolar disorder I or II;	4.8%	Presence of such conditions could potentially confound the research.
No history of diagnosed substance use or dependence disorder;	1.6%	Presence of such histories could potentially confound the research.
Not currently undergoing any other form of cognitive rehabilitation;	3.2%	An alternative form of cognitive rehabilitation could confound the research.
Not currently experiencing an active relapse;	3.2%	Ethically, it would not be appropriate to ask individuals to participate in the program if they were experiencing an active relapse. Moreover, an active relapse could confound the research.
Are a resident of the Republic of Ireland;	1.6%	Residency in Ireland is a practical requirement in relation to the feasibility and acceptability of the trial in the context of a country whose healthcare is provided by the Health Service Executive and more specifically, COB-MS delivered through CORU-registered occupational therapists.
Not living with cognitive impairment that would affect reliable participation or capacity to give informed consent;	0%	Cognitive impairment to such extent would impede participants’ ability to reliably participate in, engage with and feasibly implement the program, which is fundamental to its design. Moreover, impairment to such extent could confound the research.
Not incarcerated or institutionalized; and	3.2%	Incarceration or institutionalisation could prohibit an individual’s ability to participate in and engage with the program.
Not living with organic brain damage (unrelated to MS).	1.6%	Presence of such conditions or damage could potentially confound the research.

### Materials

An Olympus Digital Voice Recorder WS-852 was used to record the telephone interviews. The semi-structured interview guide (see Table 2) was developed in light of the SWAT’s aim of exploring the experience of being deemed ineligible; and was further informed by: researcher observation during the recruitment process; expert review; and the host trial’s embedded patient researcher (i.e. *patient & public involvement*). The COREQ checklist [18] provided a framework for study development and guidance for reporting of results.

**Table 2:** Interview schedule

1. Can you tell me how you felt when you were told that you were not eligible to participate in this study? • Prompts: ‘Can you tell me a bit more about each of these feelings’ and ‘Why you felt like that (for each emotion)’ 2. Was the reasoning behind your ineligibility to participate easy for you to understand? • Prompt: If not, get the person to tell you a bit more re this. 3. Were there any eligibility criteria that you thought might be too limiting or unnecessary? • Prompt: ‘Were you concerned that you might not be eligible in advance of being told?’ 4. Can you tell me about any past experiences you might have had in participating in research? • Did you find it a positive or negative experience? • Were you ever deemed ineligible for research before? 5. Has your experience of being ineligible for this study changed your perception or attitude toward research? • Prompt: If yes, ‘how so?’ 6. Do you think you would be willing to participate in research in the future? • Prompt: If no, ‘why not?’ 7. What did you think of the manner in which we advertised the COB-MS study? 8. What did you think of the manner in which we communicated your ineligibility? • ‘What do you think would be the best way to go about this in future research?’ 9. We often find it difficult to recruit people to participate in research studies like this, so what advice would you give as to how we might best do this in the future? • Prompt: Ask them specific unto eligibility (e.g. How can eligibility criteria or programs like this be improved so that they can help recruitment to programs like this?) 10. Finally, we asked you earlier about your feelings regarding being recruited and later determined ineligible to take part in this research. What do you think of the value of making participants feel appreciated in being recruited to a program like this and how do you think that can be best achieved?	

### Procedure

A subset of individuals deemed ineligible for the host trial consented and were telephone interviewed regarding potential impacts of being deemed ineligible. Participants were called and interviewed at a time previously agreed with the researcher. Specifically, an experienced researcher conducted both the interviews and the data analysis. Two participants were then asked to member-check these analyses (i.e. wherein participants feedback in light of reviewing the data, analyses and interpretations for the purpose of validation), confirming consistency between participant perspectives and the analyses; thus, allowing for confirmation of trustworthiness of findings reflecting the experiences of those deemed ineligible.

## Results

Eight (*N* = 8) participants consented and participated in the semi-structured telephone interviews during the summer of 2020. The interviews lasted between 21 and 44 min (M = 33:38 min). Participant characteristics are presented in Table 3. To achieve the required eight participants, overall, 11 individuals were invited to participate in the telephone interviews: one declined and two did not respond.

**Table 3:** Participant characteristics

Pseudonym	Sex	Residential location	Age	Reason for ineligibility
Amanda	F	Rural	42	History of bipolar disorder
Barbara	F	Suburban	37	Currently undergoing other form of cognitive rehabilitation
Colin	M	Rural	38	Institutionalized
Deborah	F	Urban	42	Does not have cognitive difficulties (i.e. per MSNQ)
Eva	F	Urban	58	Two reasons: Ongoing *institutionalisation* for *substance abuse*
Frances	F	Suburban	59	Currently experiencing an active relapse
Grace	F	Suburban	64	Neurologic history other than MS (i.e. vascular dementia)
Harry	M	Urban	60	Not a resident of the Republic of Ireland

**Fig. 1:**
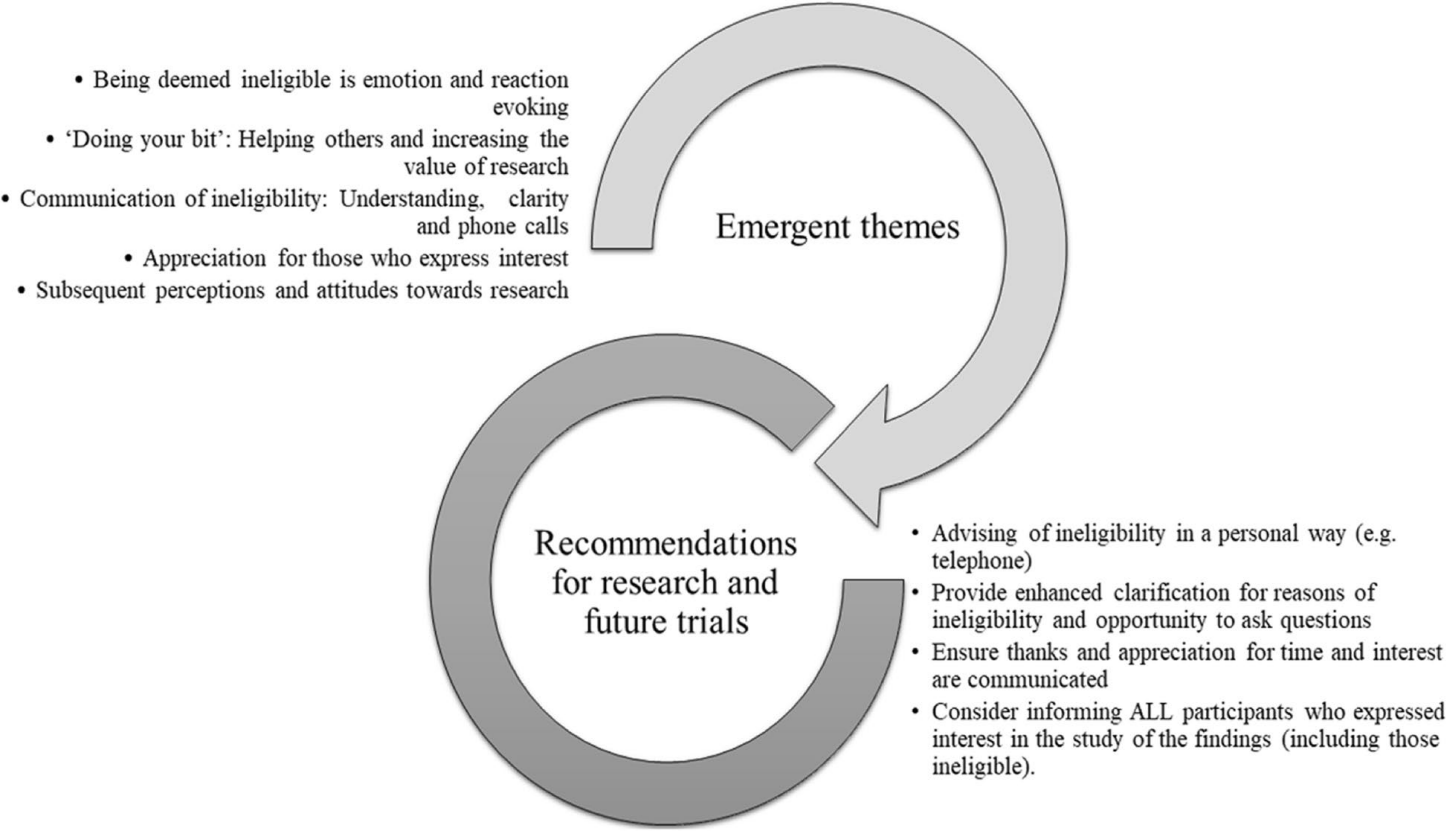
Implications of the emergent themes

Results from the qualitative interviews indicated that a majority of the individuals interviewed were accepting of the decision and understanding of the rationale for their ineligibility, albeit at differing levels of comprehension and with differing emotions and reactions. Recommendations were also made for how ineligibility can be best relayed in the future. Finally, the impact of being deemed ineligible for a study on perceptions and attitudes towards research was explored. Specifically, five themes emerged from the interviews regarding the experience of being deemed ineligible, with their implications presented in Fig. 1.

### Theme 1: Being deemed ineligible is emotion and reaction evoking

Participants reported a diverse array of emotions and reactions, including those that were positive (e.g. Deborah), negative (e.g. Amanda) and those that were void of slant (e.g. ‘it didn’t bother me – Frances; and ‘I didn’t really feel *any* way’ – Barbara). However, one emotional response that was present for a vast majority of participants, even those that were accepting of the criteria’s rationale, was that of disappointment. For example, according to Harry:

“*It was disappointing a bit… here I was trying to be involved with the cognitive study and was told ‘well, thank you for asking’*.”

Grace also found it disappointing – labelling the emotion as disheartening and deflating:

“*It’s disheartening to go forward for research and then to be told, ‘Well no, because you don't just have MS’. You know? I'd love to just have MS and nothing else. So, it is disheartening… and it's deflating as well… I just thought, well, maybe something about me can help somebody else and all of a sudden, I wasn't of interest because I didn't just have MS. So yeah, it's a bit… a bit upsetting, you know, to think that that’s another hole I don't fit into…It's not easy having different complaints. There's no point calling them illnesses because I have to live with them so… To put yourself forward and then… it's a bit of a knockback*.”

Grace’s disappointment may stem from the perspective of not fitting in – not being included due to box-ticking. She indicates that she would ‘love’ to *only* have MS, as if the reasoning behind her ineligibility somehow reflected her own doing, as if she chose to have a comorbidity – which, of course, was not the case. She expressed this in a joking manner, with wry sarcasm. She knows her ineligibility was not her fault; but, nevertheless, she felt left out – another hole she did not fit into.

According to Deborah:

“*I suppose I was interested in doing this study, so I was a little disappointed that I wasn't able to do it; but also, on the other side of it I was happy that I wasn't bad enough to do it either, you know? So there was a bit of… different emotions there, I suppose… I suppose it was a bit of a shock that I wasn't eligible, like… and I suppose I wasn't expecting a ‘no’; and yet, it was a very mixed reaction. As I said to you there, like I wasn't expecting it at all. So I was kind of like, ‘oh God, I can't do it now’ and then it was ‘Oh my God, I'm not bad enough to do it now’, so, that’s good! You know? It was a really mixed kind of emotion!*”

In the last response, Deborah was indeed happy by the news, even though she was also a bit disappointed. It seemed from her interview that this disappointment, to some extent, may have stemmed from the ‘shock’ she received at hearing of her ineligibility (i.e. contrary to Eva, Grace and Colin – who had been concerned about their ineligibility prior to being told). Indeed, the same shock or ‘surprise’ may have influenced Amanda’s negative reaction to being told of her ineligibility:

“*I have a kind of a ‘I don't care attitude’… I don't care. I said fine…It’s just another little thing to forget about… You shrug it off… [but] I found it a surprise to be honest. I probably… just when I was told that I couldn't partake, I found it a bit confusing because I'm still a person with MS so, it doesn’t really matter what other illness I have; but, I couldn't take part. You know, I just felt that you’re excluded and that’s it and you didn’t fit what they want; so, yeah, I just thought it was a bit ridiculous, to be honest. I wouldn't have taken it as an insult – I would have found it a bit daft from their point of view that they can cut people out just because they suffer from a different illness… So I just found it a bit silly really, from the point of view of the people organizing the survey…That that's how they think. You know, that that I can’t fit in.*”

Though Amanda’s response was interpreted as containing elements of annoyance, indifference and, potentially, some resentment (i.e. in addition to surprise), not all negative responses and reaction manifested in this manner – that is, directed at trial decision-makers. For example, on the other hand, Eva was ‘cross’ with herself, in light of being declared ineligible for reasons of alcoholism. According to Eva:

“*Oh, I knew the minute it mentioned alcohol. But I made a point in saying that I wouldn’t be drinking every day, but I didn't want to give the impression that I've been off it for 20 years. Once I said I'm still active, ‘uh oh’, gone. [laughs] I mean, I didn't sit at home and cry about it or anything. I just, as I say, I was frustrated with myself*.”

As evidenced from these responses, one common factor that arose within many was the notion of not ‘fitting in’, which may have latently impacted the feeling of disappointment in some participants, despite their positive attitudes towards research (see Theme 5); or even happiness (e.g. Deborah) and relief (e.g. Frances) from being deemed ineligible.

### Theme 2: ‘Doing your bit’: helping others and increasing the value of research

The notion of ‘helping others’ and increasing the value of research emerged as the second major theme from the interviews, representing both a reason for why they initially expressed interest in participating, as well as why they applied despite acknowledging that they probably wouldn’t be eligible. For example, Colin noted:

“*I half-thought I wouldn't qualify given me own circumstances anyway; but I said, I can try and help in some fashion, you know?…I suppose I was a bit disappointed because I thought, well, this is an opportunity to try and help… They’re doing their bit, if you get me? I think it should be rewarding, as in a sense of a kind of achievement kinda thing, like ‘yeah, I participated in this study!’ That was how I kind of felt in these other studies I done… when I done my bit, like, if you get me, for the greater good. The way… that's how I feel about it because… like, I really appreciated being on the [specific trial] because I think it's a wonder drug, but it wouldn't be here only for people participating in the trial… Like I find that, you have to give something back to get something better down the road where you might need it*.”

Colin’s quote is interesting to consider because it addresses his emotional reaction of disappointment (as addressed in the first theme), his understanding of the eligibility criteria (see Theme 3 below), as well as his goal of helping others or ‘doing your bit’. Similarly, Grace discusses the concept of helping others in reference to the importance of research and making people aware of the value of participating in research:

“*You're putting yourself forward in the hope that it will help somebody else. That’s the way I would look at research; like if they can find something in me, you know? When I die I want my brain to go to… some kind of research about brain disease, in the hope that it would help somebody else… if you can learn one thing from one patient, that’ll help towards the future… Do people realize why research is done? Do they see the benefit in… it's a bit like being an organ donor and some people don't see the value in it. Yeah, so maybe to hit – not their conscience, but… just to make them realize that it's for the benefit of others that, you know, it may help them*.”

Likewise, Frances, Harry and Barbara all addressed helping others as a reason for initially wanting to get involved. For example, according to Barbara:

“*For people with MS… any kind of research into how things could be improved for people's lives is useful and I feel like I should make the effort to try and give any kind of insight that I might have that would help*.”

Overall, the theme of ‘doing your bit’ addresses some important characteristics of wanting to help others. There was an element of obligation felt in some cases, such as in Barbara’s response, in terms of feeling like she should ‘make the effort’. The concept of feeling like one ‘should’ help might be explained in terms of genuine altruism, given that these individuals have the shared experience of living with MS; thus, knowing from their own lives that any form of help might be useful to others. It might also be explained in terms of the opposite – that there is a self-preservation-like function to helping, in that if one ‘does their bit’ now, they might experience some benefit later. For example, as Colin indicated – ‘you have to give something back to get something better down the road where you might need it’. Similarly, it may just be a combination of both with respect to helping for altruistic reasons, but also gaining from that act of helping – be it from gaining something later, as suggested in the last example, or simply enjoying the feeling that one has contributed in some kind of meaningful, purposeful manner, as suggested by Grace.

### Theme 3: Communication of ineligibility: understanding, clarity and phone calls

The third theme, ‘communication of ineligibility’, consists of three sub-themes: (1) understanding of ineligibility and related criteria; (2) the need for clarity; and (3) the positive impact of a phone call – all of which describe the manner in which ineligibility, further information and a personal connection are relayed to individuals who express interest in participating.

#### Understanding of ineligibility and related criteria

In a majority of the interviews, participants indicated that they understood – to varying degrees – both the rationales for their exclusion and for eligibility criteria in general. For example, according to Grace:

“*Yeah, it's easy to understand. Like if you're looking for something specific, then you're looking for something specific. You just ‘muddied the waters by making it too wide’, you know, because if you were to do research on me for MS and then something pops up when you turn around, ‘Well, I've never heard of that thing and MS before’ and suddenly think no, because that's got nothing to do with that MS. No, it was easy to understand*.”

Likewise, Colin stated:

“*In order for the trial to be effective, I suppose that you have to be able to tick off these boxes, like there's no point being wishy washy about it… for this trial to be accurate you have to be x, y and z and I thought it’s reasonable to have an effective trial that you have to tick all these boxes… I didn't find that it was… umm, ‘Oh, this is very tough to take’, you know, for the qualifying criteria. It's more realistic. … Like, for talk’s sake, if I qualified, is that not going to put your test results skew-ways? Because it's not actually… my symptoms are MS-related, so is that going to put the whole… the results off if you broaden the scope that I could qualify? So, you have to take that into consideration to get an accurate result*.”

Both Colin and Grace exhibited understanding of the rationale for eligibility criteria and their potential impact on the research. However, it also implies that they understand that the intervention (potentially) on offer is part of a research study and that for the intervention to be evaluated properly – for future use – specific guidelines must be followed. With that, consistent with the following sub-theme, it is also suggested that perhaps greater clarity is necessary for conveying the importance of such guidelines.

#### The need for clarity

Though participants, for the most part, exhibited understanding of both the rationales for their exclusion and for eligibility criteria in general, in a majority of cases, this was a loose understanding at best. Furthermore, though both Frances and Colin otherwise indicated that the criteria was made clear in ‘black-and-white’ on the participant information sheets (and that perhaps not everyone read them in full), results from the interviews indicate that more clarity is necessary in explaining the rationale for eligibility criteria in order to facilitate understanding. This rationale is equally important for people who might be upset or disappointed by the notification of ineligibility, as well as those who were fine with the decision, but still would have like to have known more – even out of simple inquisitiveness, given that they did take the time to make contact and express interest in participating. Doing so subsequent to relaying notification of ineligibility might be particularly useful. For example, according to Harry:

“*You folks know exactly what you're looking for, but I kind of wish I knew more… and if the answer was the same, fine; but, it just felt like it would have been nice to have one bit of discussion about why it didn't work out… Sort of you know, like when you break up, they say ‘it's really not you, it's me’. You know? I had no idea what the difficulty was*.”

Even in a situation where happiness was part of the reaction to being deemed ineligible, greater clarity was still desired:

“*Let them know why they’re not eligible, you know? I mean, I was fine to know, ‘OK, you're not too bad for this’, you know, I'm not needed for this – which is fine. That was great, but, another way of looking at it is, ‘well, why wasn't I?’ and ‘what was the reasoning behind me not being able to do this?’… I probably would have liked to have known more about it… I didn't think loads about it, now, don't get me wrong, I wasn't bad about it or anything… So that wasn't a problem, but just to know the reason behind why I wasn't eligible, I suppose*.” - Deborah

#### Positive impact of a phone call

Once individuals who had expressed interest in the trial had been deemed ineligible, they were advised over the telephone. The manner in which this information was relayed and the positive reactions it yielded emerged as a third sub-theme of ‘Communication of Ineligibility’. Specifically, responses indicated that with phone calls, there’s no ‘messing about’ and that phone calls facilitate ‘personal connection’. According to Deborah:

“*She was so nice on the phone… She explained everything in detail and then just went through all the questions and I answered as best I could… I was happy that I was told straight away, as well. There was no messing about… it was done perfectly on the phone*.”

Likewise:

“*I thought that it was fine because there was no kinda messing or anything… Whatever way you communicated initially, you should communicate back the same way… Like if you if you were talking on the phone and then you sent me an email later about that kind of thing – well look, I just talked to you there on the phone, why didn’t you ring me back and tell me? If you begin with the email that’s fine, but once you’ve made the phone call… it’s more of a personal connection. I know it’s professional, but still it’s more of a personal connection…. A lot of [people with MS] will be living alone or they might have carers; and one phone call could make their day – might be the only contact they had all day with anybody… So, just the fact that you made the effort to tell them ‘thank you for trying. Thank you for applying’*.” – Eva

Responses suggest that the personal connection facilitated by a phone call allows individuals to hear notification of their ineligibility in a more ‘human’ kind of situation. It also provides a chance for individuals to ask questions and gain answers in the immediate, as opposed to waiting on an email or some other form of correspondence. Furthermore, it facilitates an opportunity for researchers to more clearly explain both the rationale for why an individual was ineligible for participation and why such eligibility criteria are important – as suggested in the previous subtheme.

### Theme 4: Appreciation for those who express interest

Consistent with previously discussed themes, the concept of making contact through phone calls and clarifying the rationale for ineligibility were also identified as methods for showing excluded individuals *appreciation* for expressing interest (such as in Eva’s previous quote), which was another major theme identified. In addition to researchers taking the time to ring each ineligible person to clarify the relevant decision-making, the interviews also identified three key ways in which to show their appreciation. One method of showing appreciation is through sharing the study’s findings with both participants *and* those deemed ineligible. For example:

“*It’d be lovely to find out what the research garnered and the information found out is or what other people have with their MS – what they suffer or what they have, so it would be good to learn the findings or the conclusion of the research, even if it's just even a simple email to all the participants you know, give us a bit of closure and to give us the findings. It would be great… We'd like to know the results because a lot of surveys just do that email of ‘Thank you very much’ and all of that, but you never hear what exactly was achieved*.” – Amanda

Likewise, according to Colin:

“*I did often wonder, how did that fare out? What were the results? …That would be kind of interesting, that it was actually worthwhile… like, ‘our story is complete now. These were our findings’, rather than maybe stumbling across it on the [MS society] website… So, in reality, people can say thank you and all that, but at the end of the day, you'd rather actually rather see the impact it's actually had*.”

Second, the individual will feel appreciation if they feel valued. According to Barbara:

“*I think everybody always likes to feel valued, don't they? Like personally, I have like a high regard for just basic… like good etiquette. Saying ‘thank you’ means a lot to me… I just personally think it's important to always be appreciative of things so… Just as long as people are thanked for their input, that’s just really valuable*.”

Similarly, Grace reinforces this sentiment through emphasising the importance of saying ‘thank you’:

“*If you do something, it's only manners to say ‘thank you’ for doing that. Thank you for giving your time… a simple email saying ‘thank you for putting yourself forward and we wish you well’ or whatever. It doesn’t have to be flowers and roses – just a very simple ‘thank you’*”.

On the other hand, if appreciation is not felt, there could be negative consequences for research in the future, for example, Grace warns:

“*Someone could very easily say, ‘well, that's the last time I put myself forward’… It doesn't have to… just a very simple thank you and hope that in the future that if anything would come forward… that they would still consider it, because, you know, you may have lost several people through this, who just say, ‘well, never again am I putting myself forward’, because it's not an easy thing to do either… So, yeah, just acknowledge all the people that have come forward… you may need them again*”.

### Theme 5: Subsequent perceptions and attitudes towards research

The fifth and final theme that emerged from the interviews refers to changes to perceptions and attitudes towards research after being advised of their ineligibility for the host trial. All but one of the participants indicated that being deemed ineligible had no effect on their perception of research, with the exception being Amanda, who indicated that:

“*I suppose [being deemed ineligible has changed my attitude] a little, really. I've been mindful of the fact that they mightn’t want me again … So, yeah, that does concern me… I mightn’t be eligible and if I'm not, fair enough, and move along with life*.”

Amanda’s response possibly suggests some resentment to being deemed ineligible – veiled by the notion of ‘moving along with life’; perhaps in the sense, that though she was disappointed, upset and even concerned about implications for her ineligibility for future research, she would cope with this and move on with her life, even if she does not totally accept it.

Despite this, *all* participants indicated that they would indeed look to take part in future research, with a vast majority communicating a very positive attitude. For example, Deborah’s positive view of research makes her likely to express interest in participating again:

“*I do believe it's brilliant what you’re doing and I believe if it's not for me, someone else has that place, you know what I mean? Someone else gets that place, so I do believe it's very good to be doing research; so, just ‘cause it wasn’t an issue for me at the time, it doesn't mean it mightn’t be in future, so I'd have no problem doing it again*.”

Similarly, Barbara sees the value of research and likewise, the value of being a part of it:

“*I think it's a valuable piece of work and I would be delighted to be able to give anything that would offer some valuable information or research material… it wouldn't make me not want to engage in something like this again, you know? I'd still be as… even after this – if something else came up that I could offer something to, I would absolutely be happy to get involved*.”

Harry also sees the value of research and participation, as well as the powerful impact they can have:

“*Actually, [being deemed ineligible for this trial] makes me a little more resolute to be involved – not in a pushy way like, ‘let me in’, but not necessarily… if it doesn't fit, that's fine, but I'm still here. I still have MS and if I can help I will; and I think that's sort of the modus operandi for many people with MS that – they want to do something… being a part of this will change and improve the cognitive treatment and discussion and understanding for medical professionals as well as the people who have MS, their supportive community, their network, all of those things. And to do that as a layperson, without a medical degree or anything like that, I think it's huge. I think it's very powerful to be told this, that's what you're doing*.”

Overall, perspectives regarding research and getting involved in the future were all positive, consistent with Theme 2’s concept of ‘doing your bit’, in that interviewees showed appreciation for the *value* of research. Such value meant a variety of things to each individual, ranging from the potential to help at least one other individual, to helping make a contribution to the community of people living with MS, to helping contribute to the field as a whole. Despite being deemed ineligible for this particular research and, thus, not being able to contribute, their perception of research’s value remained.

## Discussion

### Interpretation of results

From a phenomenological standpoint, results suggest that being deemed ineligible to take part in a research trial of an intervention for chronic illness can elicit negative emotional outcomes (i.e. disappointment), which are potentially more impactful, contextually speaking, given that such individuals are simultaneously living with at least one chronic illness (i.e. in the context of the host trial, multiple sclerosis). Results suggest that being deemed ineligible is not likely to change potential participants’ perceptions of or attitudes towards research, perhaps as a result of a desire to help others similar to themselves, which is consistent with research by Author et al. [19]. However, it can; which may be detrimental to future research wherein there are smaller pools of potential participants from which to recruit. As a result, certain protocols should be followed to help minimise or even overcome the potential for such issues.

Results from the interviews also suggest that such protocols include advising of ineligibility in a personalised way, such as over the telephone: with enhanced clarity regarding the reasoning behind the decision; providing opportunities to ask questions; and ensuring thanks and appreciation for their time and interest are communicated. Though there is a genuine dearth of research exploring the impacts of being deemed ineligible for research trials, these results are consistent with past research of similar concepts. For example, recent research [20] exploring the impact of being deemed ineligible for kidney donation suggested that upon being advised of their ineligibility, individuals interested in the study were often taken aback and recommended that such outcomes should be communicated to those deemed ineligible thoroughly and with clarity; and at a time they could be mentally prepared and have an opportunity to ask questions. Past research also indicates that showing appreciation is an important aspect of working with human participants in research. For example, an important implication of appreciation (and respect) in the context of research participation is the need to express that participants’ time, effort and assistance are valued; and that the participants are viewed as valuable partners in the research process [21]. Similarly, it is important to inform participants of the results of studies in which they participate [22, 23]. Though these studies deal primarily with *participants*, it is reasonable to suggest that these impacts similarly relate to those who offer their time, effort, and assistance through their expression of *interest in participating*, as evidenced through the current study’s data.

Notably, though not an emerging theme, the issue of eligibility criteria potentially being too ‘restrictive’ also arose, consistent with recent research – in the sense that they keep individuals within specific sub-cohorts from taking part and engaging in a potentially beneficial trial, thus, altering the population being studied, in subtle ways, from that the trial was designed to help [24]. For example, as in the case of Amanda, restricting individuals with bipolar disorder yields the trial untested with respect to such comorbidity. Indeed, her perspective is consistent with that of Witham and colleagues [24]: “*I’m still a person with MS so, it doesn’t really matter what other illness I have.”* Though it is acknowledged that the sample pool eligible to participate may not be fully representative of the wider cohort, eligibility criteria are necessary for diminishing the likelihood of confounds that may both impact the ability to confidently observe treatment effects and lead to type I/II errors; otherwise, potential benefits of the trial will not be discernible, in which case, no one benefits. Indeed, with appropriate eligibility criteria in place, if a treatment is found to be of benefit, then it will be made available to those previously deemed ineligible after the trial. With that, it is acknowledged that the *appropriateness* of such criteria is key. That is, each and every criterion should serve a practical purpose consistent with best practice for research design, rather than simply easing administration of the trial (e.g. unless fluency in or residency of a particular language or country plays a genuinely practical role or serves a design-based purpose, researchers should reconsider its inclusion). Thus, it is recommended that future research focuses deeper on the rationale for each criterion included in their list for eligibility (i.e. assess whether the issue represents a genuine potential for confound), so as to not exclude potential participants that could otherwise meaningfully participate and engage with a trial.

### Strengths & limitations

There were a number of strengths of the current research’s methodology – implemented to reduce and, if possible, remove the potential for limitations. For example, the selection of participants to be invited for interview was randomly selected from the pool of individuals ineligible for the host trial and was focused on representation across reasons for ineligibility, by mapping back to the host trial. However, a limitation in any research is the potential that those who agree to take part are only a subset of the larger cohort – which is of further interest to consider given the nature of the current study’s focus on recruitment and ineligibility. Though this limitation is acknowledged, use of randomisation to select interviewees helped minimise this type of bias. It is also worth noting that only one participant declined to be involved in this SWAT. Another strength of the SWAT was the rigour and approach to the analysis, which utilised an IPA approach and member-checking. Finally, one further limitation necessary to consider was the nature of this study as a SWAT being conducted within only one host trial; and so, replication of the SWAT being embedded in multiple scenarios or trials would enable better understanding of whether participants’ experiences and perspectives are a function(s) of the trial, multiple sclerosis or some other variable not relayed within the data.

## Conclusions

The current SWAT yielded an interesting narrative-like accumulation of findings, resulting specifically from a phenomenological exploration of participants’ experiences of being deemed ineligible to participate in a RCT. Though some research has previously examined ineligibility, it has been limited to, for example, ineligibility rates, reasons, and impacts on sample sizes and allocation [1–7]; thus, given the genuinely novel findings elicited from the current research, future research is necessary to: corroborate the findings of the current SWAT; further assess the impact of the emergent themes; investigate potential gaps in the current research through identifying other potentially important factors for investigation; and assess the effects of implementing the proposed solutions (e.g. those regarding clarity and appreciation) in future research with human participants. Given the limited body of extant research on the impacts of being deemed ineligible on the individual, alongside the qualitative nature of the current study, caution must be exercised in how these results are applied, given the lack of generalisability. Nevertheless, the current SWAT engages a novel focus of research and yields a number of useful recommendations for investigating impacts of ineligibility, as well as how to improve extant protocols for engaging cases of ineligibility. Thus, while caution is indeed recommended, the results do provide both interesting recommendations for future eligibility protocols and an interesting starting point for future research on the impacts of being deemed ineligible for research trials. Overall, the current research recommends that future trials advise of ineligibility over the telephone, with researchers prepared to provide greater clarity regarding rationale and opportunities to ask questions; and ensure that thanks and appreciation are extended to those ineligible for their time and interest.

## Data Availability

Data supporting the results and analyses presented in the paper can be found at the Irish Qualitative Data Archive at dri.ie “‘How ineligibility impacts potential participants and future participation in research trials’”.

## Footnotes

1 * This SWAT was conducted as part of a larger trial of the Cognitive Occupation-Based program for people living with multiple sclerosis [25], from here on referred to as ‘the host trial’.
